# A downstream molecule of 1,25-dihydroxyvitamin D3, alpha-1-acid glycoprotein, protects against mouse model of renal fibrosis

**DOI:** 10.1038/s41598-018-35339-x

**Published:** 2018-11-26

**Authors:** Jing Bi, Hiroshi Watanabe, Rui Fujimura, Kento Nishida, Ryota Nakamura, Shun Oshiro, Tadashi Imafuku, Hisakazu Komori, Masako Miyahisa, Motoko Tanaka, Kazutaka Matsushita, Toru Maruyama

**Affiliations:** 1 Department of Biopharmaceutics, Graduate School of Pharmaceutical Sciences, Kumamoto University, 5-1 Oe-Honmachi, Chuo-ku, Kumamoto, 862-0973 Japan; 2 Program for Leading Graduate Schools “HIGO (Health life science: Interdisciplinary and Glocal Oriented) Program”, Kumamoto University, 5-1 Oe-Honmachi, Chuo-ku, Kumamoto, 862-0973 Japan; 3 Center for Clinical Pharmaceutical Sciences, School of Pharmacy, Kumamoto University, 5-1, Oe-honmachi, Chuo-ku, Kumamoto, 862-0973 Japan; 4 Department of Nephrology, Akebono Clinic, 1-1 Shirafuji 5 Chome, Minami-ku, Kumamoto, 861-4112 Japan

## Abstract

Renal fibrosis, the characteristic feature of progressive chronic kidney disease, is associated with unremitting renal inflammation. Although it is reported that 1,25-dihydroxyvitamin D3 (1,25(OH)2D3), the active form of vitamin D, elicits an anti-renal fibrotic effect, its molecular mechanism is still unknown. In this study, renal fibrosis and inflammation observed in the kidney of unilateral ureteral obstruction (UUO) mice were reduced by the treatment of 1,25(OH)2D3. The plasma protein level of alpha-1-acid glycoprotein (AGP), a downstream molecule of 1,25(OH)2D3, was increased following administration of 1,25(OH)2D3. Additionally, increased mRNA expression of *ORM1*, an AGP gene, was observed in HepG2 cells and THP-1-derived macrophages that treated with 1,25(OH)2D3. To investigate the involvement of AGP, exogenous AGP was administered to UUO mice, resulting in attenuated renal fibrosis and inflammation. We also found the mRNA expression of CD163, a monocyte/macrophage marker with anti-inflammatory potential, was increased in THP-1-derived macrophages under stimulus from 1,25(OH)2D3 or AGP. Moreover, AGP prevented lipopolysaccharide-induced macrophage activation. Thus, AGP could be a key molecule in the protective effect of 1,25(OH)2D3 against renal fibrosis. Taken together, AGP may replace vitamin D to function as an important immune regulator, offering a novel therapeutic strategy for renal inflammation and fibrosis.

## Introduction

Chronic kidney disease (CKD), which leads to progressive and irreversible kidney destruction, currently affects 10% of the population worldwide. Limited donor organ availability and growing healthcare costs highlight an unmet medical need for effective therapeutics to treat CKD. Renal fibrosis, the central feature of CKD, is associated with consistent renal inflammation or inadequate homeostatic repair responses^1,2^. Kidney inflammation involves both immune cells and activation of intrinsic renal cells, leading to the release of pro-fibrotic cytokines and growth factors that serve as major driver of persistent fibrosis^3^. Although many molecules and pathways that drive renal fibrosis and inflammation have been identified in experimental models^4–6^, our knowledge of endogenous factors that may limit renal fibrosis remains inadequate.

In patients with CKD, active vitamin D deficiency is associated with a reduction of renal mass^7^ and an increased risk of mortality^8^. Along with the classical role in bone homeostasis, active vitamin D is also involved in immune modulation^9^. A number of active vitamin D analogues are known to function as vitamin D receptor activator (VDRA). The renoprotective effect of VDRA exogenously administered against renal injury has recently been established. Indeed, 1,25(OH)2D3 was reported to protect against various kidney injuries including anti-Thy-1.1 nephritis^10^, nephrectomy^11^ and diabetic nephropathy^12^. In addition, 22-oxacalcitriol was found to protect against glomerulonephritis in rats^13^, and paricalcitol reduced proteinuria in CKD patients^14^. Specifically, paricalcitol attenuated renal fibrosis by directly inhibiting TGF-β-SMAD transduction^15^. It is conceivable that VDRA may elicit a renoprotective mechanism due to its multitude of actions. However, it is still unclear which downstream molecules of VDRA lead to an immunomodulatory effect.

Looking into the immunomodulatory effects of VDRA, we focused on alpha-1-acid glycoprotein (AGP). This acute-phase protein, also known as orosomucoid, exhibits a variety of activities including anti-inflammation and immune modulation. AGP is primarily synthesized in the liver and in some extra-hepatic cells, including monocytes and macrophages^16^, and its plasma concentration is elevated 2 to 5-fold under inflammatory conditions^17,18^. Recently, we demonstrated that AGP inhibited the production of IL-6 and TNF-α and induced CD163, a specific marker that possess anti-inflammatory potential expressed predominantly on monocyte/macrophage, *via* the TLR4 pathway^19^. Additionally, AGP protects against mouse models of ischemia/reperfusion^20^ and puromycin-induced renal injury^21^ by preventing apoptosis and inflammation. Besides, AGP plays a pivotal role in the anti-fibrotic effects of imatinib in pulmonary fibrosis mouse model^22^. Therefore, it is speculated that AGP may also protect against renal fibrosis. Importantly, in an *in vitro* system using U937 cells, a human monocyte cell line, Gemelli *et al*. found that AGP production was enhanced by VDRA through vitamin D receptor (VDR)^23^. Taken together, AGP may act as a downstream molecule in the anti-renal fibrotic effects of VDRA.

To clarify this issue, we examined the anti-fibrotic activity of 1,25(OH)2D3 using a mouse renal fibrosis model. We examined the effects of 1,25(OH)2D3 on AGP induction both in mice and *in vitro*, and the anti-renal fibrotic effects of AGP and its possible mechanism.

## Results

### 1,25(OH)2D3 protects against kidney fibrosis and inflammation after unilateral ureteral obstruction

**Figure 1:**
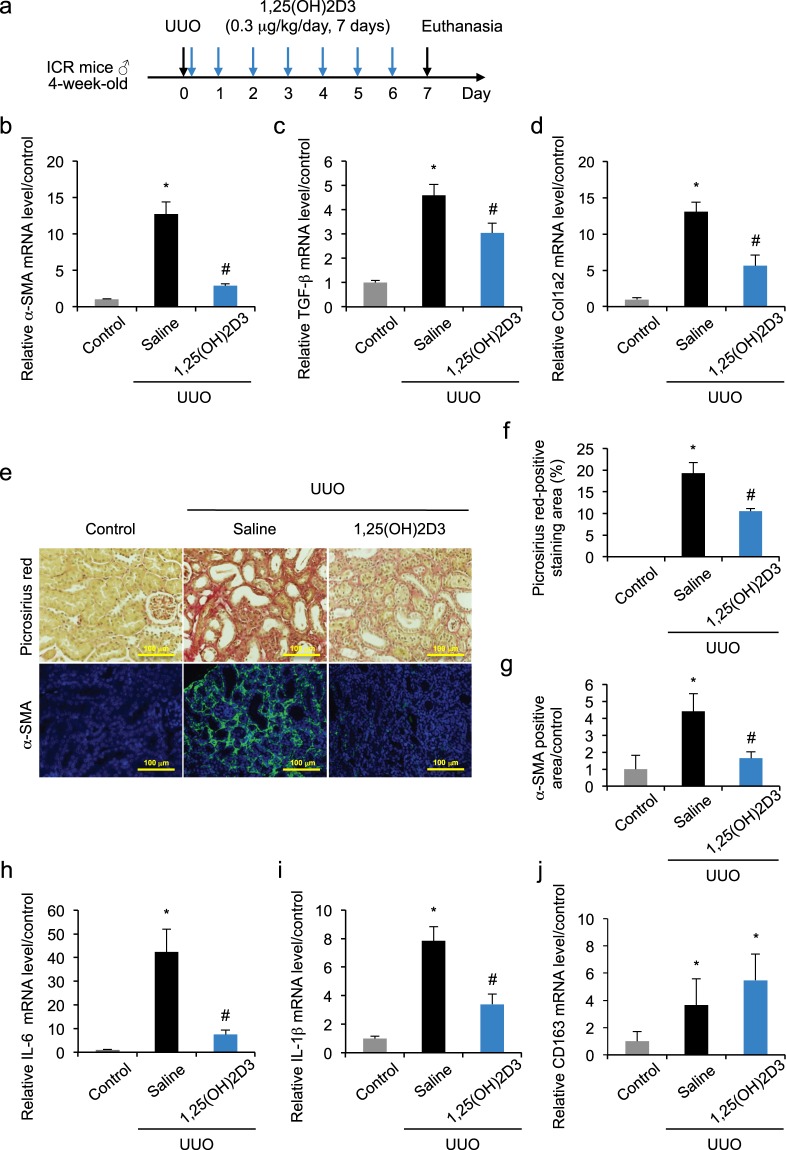
1,25(OH)2D3 protects against kidney fibrosis and inflammation induced by UUO. (**a**) 1,25(OH)2D3 was injected intraperitoneally once-daily from day 0 to day 6 after the UUO treatment. (**b**–**d**) UUO up-regulated mRNA expression of α-SMA, TGF-β and Col1a2 at day 7, and this up-regulation was reduced following 1,25(OH)2D3 treatment. (**e**) Kidney fibrosis was determined by Picrosirius red staining (upper); the red color in Picrosirius red staining is described in (**f**). Double immunofluorescent staining of α-SMA (green) with DAPI (blue) (lower); the green fluorescence was quantified in (**g**–**i**) UUO up-regulated mRNA expression of IL-6 and IL-1β at day 7, and this up-regulation was suppressed by 1,25(OH)2D3 treatment. (**j**) CD163, a monocyte/macrophage marker with anti-inflammatory potential, increased in UUO-pathology and this was enhanced by 1,25(OH)2D3 treatment. Scale bar, 100 μm. *P < 0.05 compared with control; ^#^P < 0.05 compared with saline; n = 6–10. Data are presented as mean ± SE.

Renal fibrosis was induced in a mouse model of UUO. To determine the treatment scheme of 1,25(OH)2D3, we firstly examined the changes in the expression of major fibrotic markers, α-SMA, TGF-β and Col1a2 in the obstructed kidneys 7 days after UUO treatment (Fig. 1a). Real-time PCR demonstrated significantly increased mRNA expression of α-SMA (Fig. 1b), TGF-β (Fig. 1c) and Col1a2 (Fig. 1d) in the UUO-treated kidneys compared with the kidney from healthy control mice at day 7. Next, we administered 1,25(OH)2D3 once-daily (0.3 μg/kg, *i.p*., from day 0 to day 6) for the following seven days to the UUO mice. Compared with saline-treated UUO mice, mRNA expression of α-SMA, TGF-β and Col1a2 was significantly suppressed by 1,25(OH)2D3 treatment. Renal fibrosis was evaluated by Picrosirius red staining for collagen (Fig. 1e,f) and immunofluorescent staining of α-SMA in myofibroblasts (Fig. 1e,g). These data demonstrated that 1,25(OH)2D3 treatment reduced renal fibrosis and decreased α-SMA positive areas (Fig. 1f,g) by day 7. In addition, 1,25(OH)2D3 treatment suppressed the increased hydroxyproline content in the obstructed kidney (Supplementary Fig. S1).

Next, we examined changes of inflammatory and anti-inflammatory markers in the UUO-induced renal fibrosis. The mRNA expression of IL-6 (Fig. 1h) and IL-1β (Fig. 1i) in obstructed kidneys showed a significant increase, and this increase was significantly suppressed by 1,25(OH)2D3 treatment. Protein level of IL-6 and IL-1β in the obstructed kidney were also suppressed by the treatment of 1,25(OH)2D3 (Supplementary Fig. S2). Additionally, mRNA expression of CD163, a monocyte/macrophage specific marker on cells that possess anti-inflammatory potential, showed a slight, but not significant, increase in saline-treated UUO mice compared with control mice. The mRNA expression level of CD163 was further increased in 1,25(OH)2D3-treated UUO mice compared with saline-treated UUO mice (Fig. 1j). Therefore, 1,25(OH)2D3 reduced markers of inflammation and fibrosis in mouse model of obstructive nephropathy. In these experiments, no significant changes in fibrosis (α-SMA, TGF-β and Col1a2 expression) and inflammation (IL-6 and IL-1β expression) were observed between the kidney in healthy control mice and the non-obstructed contralateral kidney in UUO mice (data not shown). Therefore, kidneys in healthy control mice were used in the following experiments as a control.

### 1,25(OH)2D3 induced alpha-1-acid glycoprotein (AGP) expression in healthy and UUO mice, HepG2 hepatocytes and THP-1-derived macrophages

**Figure 2:**
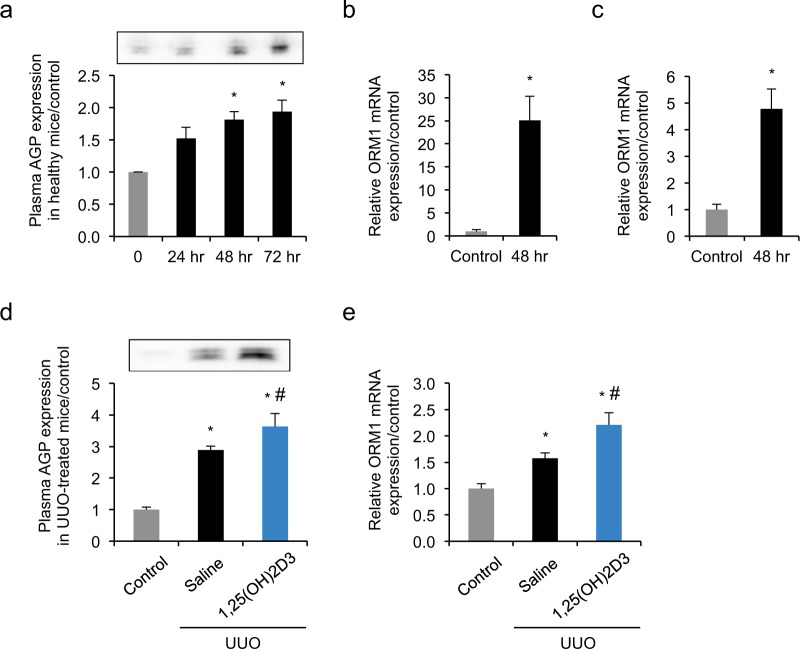
1,25(OH)2D3 induced AGP expression in healthy and UUO mice, hepatocytes and THP-1-derived macrophages. (**a**) 1,25(OH)2D3 (0.3 μg/kg) was injected intraperitoneally to healthy mice at 0, 24 and 48 hr. Western blotting showed plasma protein level increased by approximately 2-fold and reached a plateau at 48 hr. mRNA expression of ORM1 was up-regulated in (**b**) HepG2 cells or (**c**) THP-1-derived macrophages following incubation with 1,25(OH)2D3 (100 nM). (**d**) 1,25(OH)2D3 (0.3 μg/kg) or saline was injected intraperitoneally once-daily from day 0 to day 6 after the UUO treatment. Plasma AGP expression was determined by Western blotting at day 7. Plasma was collected from healthy mice, saline-treated UUO mice or 1,25(OH)2D3-treated UUO mice. (**e**) 1,25(OH)2D3 (0.3 μg/kg) or saline was injected intraperitoneally once-daily from day 0 to day 6 after the UUO treatment. At day 7 after UUO, mRNA expression of hepatic ORM1 was determined by quantitative RT-PCR. Liver was collected from healthy mice, saline-treated UUO mice or 1,25(OH)2D3-treated UUO mice. *P < 0.05 compared with 0 hr or control; n = 3–5. Data are presented as mean ± SE.

To investigate whether the treatment of 1,25(OH)2D3 induces AGP expression *in vivo*, we injected the same dose of 1,25(OH)2D3 (0.3 μg/kg) intraperitoneally to healthy mice at 0, 24 and 48 hour (hr). Western blotting confirmed that the plasma AGP level increased by approximately 2-fold and reached a plateau at 48 hr in healthy mice (Fig. 2a), demonstrating that AGP was induced by 1,25(OH)2D3 treatment *in vivo*.

Because AGP is synthesized in the hepatocytes and extra-hepatic cells including macrophages, we next evaluated mRNA expression of AGP following the 1,25(OH)2D3 treatment in hepatocyte cell line (HepG2) and phorbol 12-myristate 13-acetate (PMA)-induced THP-1-derived macrophages. HepG2 cells or THP-1-derived macrophages were incubated with 1,25(OH)2D3 at a concentration of 100 nM for 48 hr. Real-time PCR demonstrated that in 1,25(OH)2D3-treated HepG2 cells, mRNA expression of *ORM1*, an AGP gene, increased 25-fold (Fig. 2b). In THP-1-derived macrophages, 1,25(OH)2D3 enhanced the mRNA expression of *ORM1* by 5-fold (Fig. 2c).

Next, we evaluated the plasma and hepatic AGP expressions with or without 1,25(OH)2D3 in the UUO mice. As a result, AGP protein expression in plasma and AGP mRNA expression in liver from UUO mice were significantly higher than those from healthy control mice. In 1,25(OH)2D3 treated-UUO mice, the further significant increases of plasma and hepatic AGP expressions were observed (Fig. 2d,e).

### AGP protects against kidney fibrosis induced by UUO

**Figure 3:**
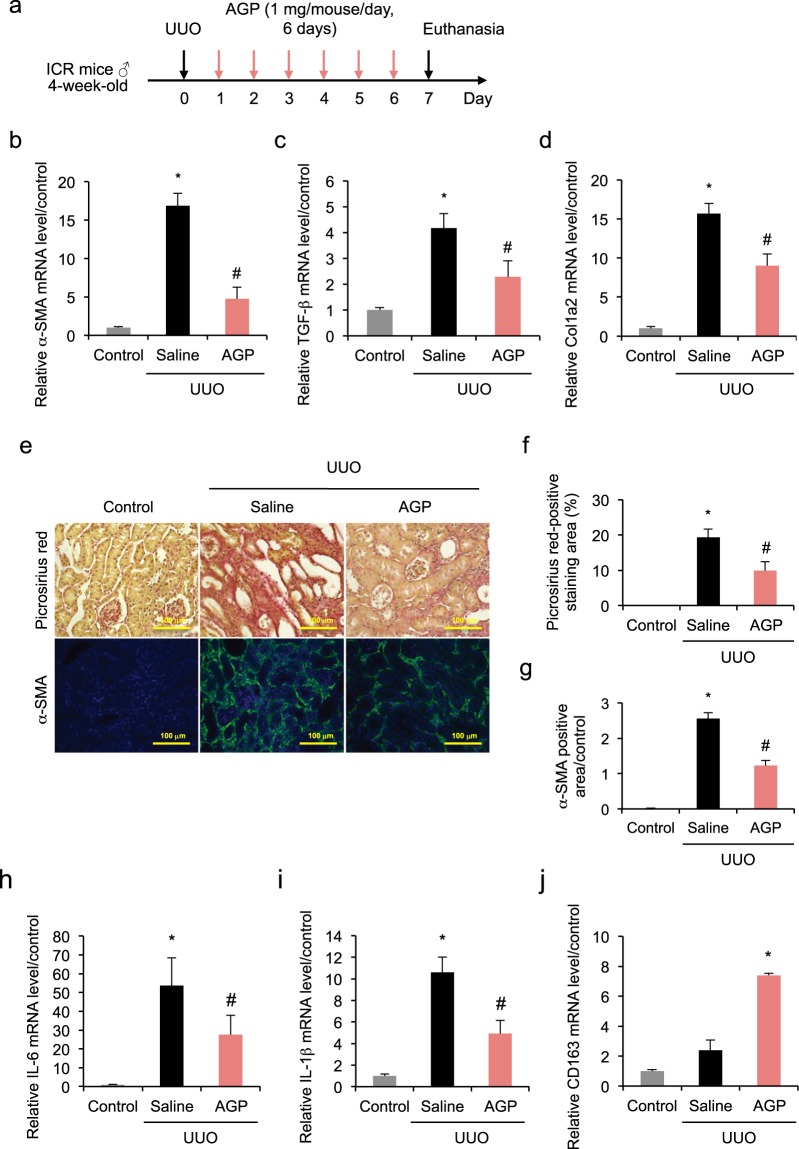
AGP protects against kidney fibrosis induced by UUO. (**a**) AGP (1 mg/mouse) was injected intraperitoneally once-daily from day 1 to day 6 after the UUO treatment. (**b**–**d**) UUO up-regulated mRNA expression of α-SMA, TGF-β and Col1a2 at day 7, and this up-regulation was suppressed by AGP treatment. (**e**) Kidney fibrosis was determined by Picrosirius red staining (upper); the red color in Picrosirius red staining is described in (**f**). Double immunofluorescent staining of α-SMA (green) with DAPI (blue) (lower); green fluorescence was quantified in (**g**–**i**) UUO up-regulated mRNA expression of IL-6 and IL-1β at day 7, and this up-regulation was suppressed by AGP treatment. (**j**) The mRNA expression of CD163 increased in UUO pathology and was enhanced by AGP treatment. Scale bar, 100 μm. *P < 0.05 compared with control; ^#^P < 0.05 compared with saline; n = 4–9. Data are presented as mean ± SE.

To explore whether an AGP concentration comparable to the plasma level of 1,25(OH)2D3-treated mice protects against renal fibrosis, the UUO mice were exogenously administered with AGP. AGP (1 mg) was injected intraperitoneally to each mouse 24 hr after UUO treatment, once-daily for the following six days (Fig. 3a). As expected, a similar effect to that elicited by 1,25(OH)2D3 was observed. Namely, increased mRNA expression of α-SMA, TGF-β and Col1a2 in the UUO-treated kidneys 7 days after the obstruction were significantly suppressed by AGP treatment (Fig. 3b–d). Assessment of the fibrotic area based on Picrosirius red staining and immunofluorescent staining of α-SMA showed AGP treatment ameliorated the fibrosis (Fig. 3e–g). In addition, AGP treatment suppressed the increased hydroxyproline content in the obstructed kidney (Supplementary Fig. S1). Consistent with this finding, the up-regulation of inflammatory markers IL-6 and IL-1β in the obstructed kidneys was suppressed by AGP treatment (Fig. 3h,i). Protein levels of IL-6 and IL-1β in the obstructed kidney were also suppressed by the treatment of AGP (Supplementary Fig. S2). Moreover, mRNA expression of CD163 increased in the obstructed kidneys (Fig. 3j), which was more evident in the AGP-treated group by comparison with the saline-treated group. As a result, AGP has a dual benefit as it reduced both fibrosis and inflammation.

### AGP plays an anti-inflammatory role in THP-1-derived macrophages with or without lipopolysaccharide

**Figure 4:**
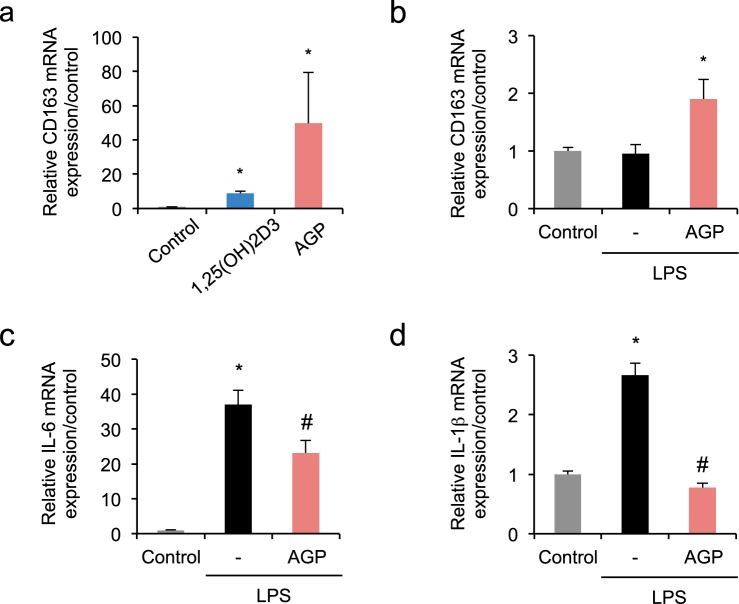
AGP elicits an anti-inflammatory effect in THP-1-derived macrophages with or without LPS. (**a**) mRNA expression of CD163 was up-regulated in THP-1-derived macrophages following 1,25(OH)2D3 (100 nM) treatment or AGP (0.5 mg/mL) treatment for 48 hr. (**b**–**d**) AGP treatment suppressed LPS-induced mRNA expression of IL-6 and IL-1β, while up-regulating CD163 in THP-1-derived macrophages. LPS (100 ng/mL) was added to the cells in the presence or absence of AGP (0.5 mg/mL) and incubated for 48 hr. *P < 0.05 compared with control; ^#^P < 0.05 compared with LPS-treated group; n = 3–4. Data are presented as mean ± SE.

To investigate the similarity of the biological actions of immune modulation between 1,25(OH)2D3 and AGP, THP-1-derived macrophages were incubated with 1,25(OH)2D3 (100 nM) or AGP (0.5 mg/mL) for 48 hr (Fig. 4a). The mRNA expression of CD163 in the AGP-treated cells increased by 50-fold. Similarly, the mRNA expression of CD163 increase was 9-fold in the 1,25(OH)2D3-treated cells. These observations suggested AGP may have a higher anti-inflammatory potential compared with 1,25(OH)2D3.

Secondly, to confirm whether the AGP-induced CD163 expression occurs under inflammatory conditions, THP-1-derived macrophages were incubated with lipopolysaccharide (LPS) (100 ng/mL) in the presence or absence of AGP (0.5 mg/mL) for 48 hr. The mRNA expression of CD163 in LPS-stimulated cells in the absence of AGP showed no significant change compared with the control group. However, a 2-fold increase of CD163 mRNA expression was observed in the AGP-treated group even in the presence of LPS (Fig. 4b). Compared with the control group, mRNA expression of IL-6 and IL-1β was up-regulated in the LPS-stimulated group, which was significantly suppressed by AGP treatment (Fig. 4c,d). AGP also tended to reduce LPS-induced IL-6 expression and significantly reduced IL-1β expression even at 24 hr (Supplementary Fig. S3).

## Discussion

Renal fibrosis is a driving force toward CKD and end-stage renal disease. Our studies on the UUO-induced renal fibrosis confirmed that (1) AGP induction was observed following 1,25(OH)2D3 treatment both *in vivo* and *in vitro* (2) AGP treatment attenuated renal fibrosis and inflammation in the UUO mice (3) AGP played an anti-inflammatory role in THP-1-derived macrophages with or without LPS stimulation. These results indicated that AGP, a downstream molecule of VDRA, could be important in the protective effect of VDRA against renal fibrosis.

Vitamin D receptor (VDR) plays a key role in the pleiotropic effects of VDRA. Consistent with the results of Ito *et al*.^24^, here we show that this treatment dosage of 1,25(OH)2D3 (0.3 μg/kg, *i.p*., from day 0 to day 6, once-daily) ameliorated renal fibrosis in UUO mice. Interestingly, we found that the equivalent dose of 1,25(OH)2D3 (0.3 μg/kg) increased the plasma level of AGP in healthy and UUO mice. As far as we know, this is the first experimental evidence in which VDRA enhances the plasma AGP expression *in vivo*. Because AGP induction is mediated through the VDRA-VDR-AGP pathway in a human macrophage cell line^23^, it is possible that a similar macrophage-associated mechanism is responsible for the plasma AGP induction after the 1,25(OH)2D3 treatment. Our studies demonstrated 1,25(OH)2D3 had the effect of inducing AGP in hepatocytes and macrophages. Particularly, the AGP induction in hepatocytes was much more pronounced than in THP-1-derived-macrophages. This observation implies that the liver is mainly responsible for VDRA-induced AGP production, while the function of macrophages is more partial and localized.

Because AGP plays an anti-inflammatory and immunomodulatory role, we hypothesized that the anti-renal fibrotic activity elicited by 1,25(OH)2D3 could be associated with 1,25(OH)2D3-induced AGP production. Our next strategy was to exogenously administer a specific dose of AGP (Fig. 3) to raise the plasma AGP concentration to an equivalent level as that induced by 1,25(OH)2D3 treatment. As a matter of fact, we found that exogenous AGP treatment showed anti-fibrotic actions by suppressing the mRNA expression of α-SMA, TGF-β and Col1a2 in the UUO-treated kidneys, consistent with the results of 1,25(OH)2D3. Likewise, AGP showed anti-inflammatory actions resembling 1,25(OH)2D3, characterized by suppressed expression of IL-6 and IL-1β, as well as up-regulated expression of CD163 in the kidney of the UUO mice. Therefore, it seems likely that AGP may be involved in anti-fibrotic effects and could function as a downstream key molecule of 1,25(OH)2D3. To ascertain the contribution of the VDRA-VDR-AGP pathway, we are currently generating AGP knockout mice. The ongoing experiments will investigate the connection between the anti-inflammatory and anti-fibrotic effect of VDRA and AGP.

At the early stage of pathogenesis at day 3 after UUO, AGP treatment did not significantly suppress the increased mRNA expression of α-SMA, TGF-β, Col1a2, IL-6 and IL-1β (data not shown). These results indicated the possibility that AGP plays its anti-fibrotic role by reversing the ongoing pathology, rather than preventing the early stage of pathogenesis. In addition, we also examined the effect of the higher dose of AGP (3 mg/mouse/day) at day 7 after UUO. However, the anti-fibrotic and anti-inflammatory effects of higher dose of AGP (3 mg/mouse/day) was lower than those of AGP (1 mg/mouse/day) (data not shown). These data suggested that the plasma level of AGP induced by 1,25(OH)2D3 (which is comparable to the AGP dose of 1 mg/mouse/day) may be an optimal concentration to the reduced renal fibrosis and inflammation. The treatment effect of lower AGP dose (<1 mg/mouse/day) would be interesting for further investigation. In addition, administration of 1,25(OH)2D3 or AGP after the induction of UUO or other kidney disease model such as 5/6 nephrectomy will further validate the effects of 1,25(OH)2D3 or AGP.

Although the plasma AGP level rises under stress conditions, this endogenous production is considered insufficient to rival consistent pathological stress. While urinary AGP secretion in healthy people is usually very low, it is detected in patients with a variety of renal diseases including nephrotic syndrome^25^ and active lupus nephritis^26^. Specifically, urinary AGP is observed from the early stage in patients with diabetic nephropathy and gradually increases with the progression of the disease, correlating with the renal damage^27^. In patients with sickle cell anemia-induced CKD, AGP in urine is detected before the onset of proteinuria^28^. This AGP leakage into the urine is one of the urine biomarkers that predict the cause of glomerular disease^29^, indicating that AGP loss may exacerbate renal injury. In this study, our strategy of supplementing AGP was effective in countering renal fibrosis (Fig. 3). In the future, we expect that AGP treatment may be applicable not only to renal interstitial fibrosis but also to other pathological conditions associated with AGP loss.

Despite the pharmacological activities of VDRAs, the risk of a hypercalcemic effect^30^ limits their further clinical application. Hypercalcemia and loss of body weight was also noted in our studies on 1,25(OH)2D3-treated UUO mice (Supplementary Table S1). In contrast, unlike 1,25(OH)2D3, exogenously administered AGP did not affect mineral homeostasis or body weight (Supplementary Table S1) although the renoprotective effects were comparable (Fig. 4). Accordingly, AGP, devoid of hypercalcemia, could replace vitamin D as a safer and better therapeutic approach for renal fibrosis.

In THP-1-derived macrophages, we noted that AGP performed anti-inflammatory effects even under LPS stimulus (Fig. 4). We have previously demonstrated that AGP up-regulated CD163 expression in both THP-1-derived macrophages and peripheral blood mononuclear cells. Then, AGP signaling through the TLR4 and CD14, the common innate immune receptor complex that normally recognizes bacterial components, was identified as a crucial stimulus that leads to an enhancement in CD163 expression^19^. In the presence of LPS, AGP could antagonize LPS signaling via TLR4. These molecular mechanisms could be involved in AGP related anti-inflammatory reaction. In contrast to our data suggesting AGP suppresses LPS-stimulated IL-6 and IL-1β expression in PMA-treated THP-1 cells at 24 and 48 hr, Boutten *et al*. and Drenth *et al*. reported that AGP potentiates IL-6 and IL-1β secretion at 24 hr^31,32^. This apparent discrepancy in IL-6 and IL-1β expression levels might be due to differences in AGP concentration. Based on our data, an incubation time of up to 48 hr with AGP significantly increased the expression of CD163 in THP-1-derived macrophages, but this increase in CD163 expression was not observed at 24 hr (data not shown). Further investigation is necessary to identify the effect of AGP on macrophage phenotype infiltrated in kidney, as the macrophage phenotype balance has been reported to be a key player in renal fibrosis^33,34^.

Traditionally, it is believed that VDRAs function through receptor signaling, in which downstream molecules play a crucial role in the biological function of VDRAs. However, it has been reported recently that VDR-independent pathways are also associated with the anti-fibrotic effect of VDRA. Paricalcitol is known to reduce renal intestinal fibrosis by directly blocking the epithelial to mesenchymal transition (EMT)^14^. Moreover, 1,25(OH)2D3 can selectively inhibit the TGF-β-SMAD signaling without activating VDR^24^. Thus, it is possible that not only the vitamin D-VDR-AGP pathway but also the VDR-independent pathway could be responsible for the anti-fibrotic effect of VDR ligands. Further research on VDR knockout mice is needed to determine the detailed function of VDR.

In conclusion, we report AGP to be a key molecule in the protective effect of VDRA against renal fibrosis. Our findings suggest AGP may function as an important immune regulator, potentially replacing vitamin D as a novel therapeutic strategy for the treatment of renal inflammation and fibrosis.

## Methods

### Purification of alpha-1-acid glycoprotein

AGP was purified from human plasma fraction V that provided by KAKETSUKEN, Kumamoto, Japan. Fraction V was dissolved in acetate buffer (10 mM) and applied to a HiTrap CM FF column (5 mL) and a HiTrap Q FF column (5 mL) on an AKTAprime Plus System (GE Healthcare, Tokyo, Japan). Bound material was subsequently eluted with acetate buffer (10 mM) containing NaCl (0.5 mol/L) at a flow rate of 5 mL/min. The eluate was dialyzed against deionized water at 4 °C, freeze-dried and stored at −20 °C. The purified protein was confirmed as AGP by Western blotting.

### Animal model of unilateral ureteral obstruction (UUO)

All animals were maintained in a room under controlled temperature with a 12 hr dark/light cycle (light 8 am – 8 pm) and freely provided food and water. All animal experiments were conducted with procedures approved by the experimental animal ethics committee at Kumamoto University.

Male ICR mice (4-week-old, Japan SLC) were randomized and anaesthetized before UUO treatment. The left ureter was ligated with 4-0 silk, and the abdomen was closed with sutures. After surgery, the mice were warmed until recovery. 1,25(OH)2D3 (Rocaltrol®INJECTION, Kyowa Hakko Kirin, Tokyo, Japan) was administered *i.p*. at a dose of 0.3 μg/kg, once-daily from day 0 to day 6 of the UUO treatment. AGP (1 mg/mouse) was administered *i.p*. once-daily from day 1 to day 6 of UUO treatment. An equivalent amount of saline was administered to the non-treated control group and the UUO group. Mice were sacrificed on day 7 after surgery.

### Histological analysis

Tissue of mouse kidneys harvested on day 7 after obstruction was fixed in 10% phosphate buffered formaldehyde for 48 hr, embedded in paraffin, sectioned (4 μm), deparaffined with xylene, rehydrated through a graded series of ethanol and then washed in water. Picrosirius red staining was performed for morphological analysis (original magnification power ×400).

### Immunofluorescence microscopy

The kidney section was solubilized with a solution containing Tris/HCl (TB, 50 mM, pH = 7.5) and a solution containing 0.1% Tween-20 (Tween-TB). Block Ace (Dainippon Pharmaceutics, Osaka, Japan) was used for blocking at room temperature for 20 min. The samples were incubated with anti-α-SMA antibody (1:100, Sigma-Aldrich, St Louis, MO) overnight at 4 °C, followed by corresponding secondary antibody Alexa Fluor® 488 goat anti-rabbit IgG (H + L) (1:200, Thermo Scientific, Rockford, IL) for 90 min at 25 °C. Samples were also treated with DAPI (Dojin Chemical, Kumamoto, Japan). The slides were then examined by light microscopy (BZ-8000; Keyence Corp., Osaka, Japan).

### Cell culture

HepG2 cells and THP-1 cells were cultured in RPMI 1640 (Sigma-Aldrich) medium with 10% fetal bovine serum (Sigma-Aldrich), 1% penicillin and streptomycin (Thermo Scientific) at 37 °C in an atmosphere containing 5% CO_2_. HepG2 cells were seeded at 1.0 × 10^5^ cells/well in 6-well plates and incubated for 24 hr, before incubated with 1,25(OH)2D3 at a dose of 100 nM for 48 hr. THP-1 cells were seeded at 1.0 × 10^6^ cells/well in 6-well plates and exposed to PMA (50 nM, Sigma-Aldrich) in the culture medium for 48 hr. The PMA-containing medium was then removed by aspiration and cells were incubated for an additional 24 hr. 1,25(OH)2D3 (100 nM) was added to each well and the cells were incubated for 48 hr. In experiments that THP-1 cells were treated by LPS, cells were stimulated with AGP (0.5 mg/mL) or LPS (100 ng/mL) and incubated for 48 hr.

### Real-Time RT-PCR

Total RNA from kidney or cells was isolated and real-time PCR measurements were carried out in a method previously described^19^. Each primer sequence is shown in Supplementary Table S2.

### Statistical analysis

Data from animal and cell studies were compared by analysis of variance followed by Tukey’s multiple comparison. All results are expressed as the mean ± SE of the indicated experiments. P value < 0.05 was considered statistically significant.

## Electronic supplementary material

Supplementary information

## Data Availability

All data generated during and/or analysed during the current study are available from the corresponding author upon reasonable request.
