# Supplementary material for: A downstream molecule of 1,25-dihydroxyvitamin D3, alpha-1-acid glycoprotein, protects against mouse model of renal fibrosis
Source: Sci Rep. 2018 Nov 26;8:17329. doi: 10.1038/s41598-018-35339-x (PMC6255841; doi:10.1038/s41598-018-35339-x)
Supplement: Supplementary file 1 — Supplementary information [file 41598_2018_35339_MOESM1_ESM.docx]

**A downstream molecule of 1,25-dihydroxyvitamin D3, alpha-1-acid glycoprotein, protects against mouse model of renal fibrosis**

Jing Bi^1,2^, Hiroshi Watanabe^1,3,*^, Rui Fujimura^1,2^, Kento Nishida^1^, Ryota Nakamura^1^, Shun Oshiro^1^, Tadashi Imafuku^1,2^, Hisakazu Komori^1^, Masako Miyahisa^1^, Motoko Tanaka^4^, Kazutaka Matsushita^4^, Toru Maruyama^1,3,*^

^1^Department of Biopharmaceutics, Graduate School of Pharmaceutical Sciences, Kumamoto University, 5-1 Oe-Honmachi, Chuo-ku, Kumamoto 862-0973, Japan and ^2^Program for Leading Graduate Schools "HIGO (Health life science: Interdisciplinary and Glocal Oriented) Program", Kumamoto University, 5-1 Oe-Honmachi, Chuo-ku, Kumamoto 862-0973, Japan and ^3^Center for Clinical Pharmaceutical Sciences, School of Pharmacy, Kumamoto University, 5-1, Oe-honmachi, Chuo-ku, Kumamoto 862-0973, Japan and ^4^Department of Nephrology, Akebono Clinic, 1-1 Shirafuji 5 Chome, Minami-ku, Kumamoto 861-4112, Japan

**Supplementary information**

**Supplementary Figure S1.** The hydroxyproline content in the obstructed kidney was suppressed by the treatment of 1,25(OH)2D3 or AGP.

Data are expressed as the mean ± SE. *P < 0.05 compared with the control mice. #P < 0.05 compared with the saline-treated UUO mice.

**Supplementary Figure S2.** The protein level of IL-6 and IL-1β in the obstructed kidney were suppressed by the treatment of 1,25(OH)2D3 or AGP.

**Supplementary Figure S3.** AGP elicits an anti-inflammatory tendency in THP-1-derived macrophages with LPS stimulus in 24 hr.

(a-c) AGP treatment suppressed LPS-induced mRNA expression of IL-6 and IL-1β, while up-regulating CD163 in THP-1-derived macrophages. LPS (100 ng/mL) was added to the cells in the presence or absence of AGP (0.5 mg/mL) and incubated for 24 hr. *P < 0.05 compared with control; #P < 0.05 compared with LPS-treated group; n=3-4. Data are presented as mean ± SE.

| **Supplementary Table S1.** Body weight and plasma calcium level profile for the UUO mice and 1,25(OH)2D3- and AGP-treated UUO mice. | | | |
| --- | --- | --- | --- |
|  | Control | 1,25(OH)2D3-treated | AGP-treated |
|  | (UUO mice) | UUO mice | UUO mice |
| Initial body weight (g) | 30.0±0.6 | 29.7±0.7 | 29.4±0.3 |
| (at day 0) |  |  |  |
| Final body weight (g) | 31.2±0.9 | *25.7±0.7 | 30.1±0.3 |
| (at day 6) |  |  |  |
| Plasma calcium level (mg/dL) | 9.1±0.3 | *14.4±0.3 | 8.7±0.2 |
| Data are expressed as the mean ± SE. *P < 0.05 compared with the UUO mice. | | | |

| **Supplementary Table S2.** Sequence of oligonucleotide primers used in the real-time PCR experiments. | | | |
| --- | --- | --- | --- |
|  | Target gene | Forward | Reverse |
|  | GAPDH | 5’-GGTGAAGGTCGGAGTCAACG-3’ | 5’-ACCATGTAGTTGAGGTCAATGAAGG-3’ |
|  | ORM1 | 5’-AGTACCAGACCCGACAGGAC-3’ | 5’-GCAAGTGAGCGAAATGCTCT-3’ |
| Human | IL-6 | 5’-CAGTTCCTGCAGAAAAAGGC-3’ | 5’-AACAACAATCTGAGGTGCCC-3’ |
|  | IL-1β | 5’-GGCTGCTCTGGGATTCTCTTC-3’ | 5’-ATTTCACTGGCGAGCTCAGG-3’ |
|  | CD163 | 5’-ACAGCGGCTTGCAGTTTCCT-3’ | 5’-ATGGCCTCCTTTTCCATTCCAG-3’ |
|  | GAPDH  ORM1 | 5’-AACTTTGGCATTGTGGAAGG-3’ 5’-ACACAATAGAGCTTCGGGAGTC-3’ | 5’-ACACATTGGGGGTAGGAACA-3’  5’-ATATCTGGCCTTTTGGCATAGA-3’ |
|  | α-SMA | 5’-AGCCATCTTTCATTGGGATGG-3’ | 5’-CCCCTGACAGGACGTTGTTA-3’ |
| Mouse | TGF-β | 5’-GGATACCAACTATTGCTTCAGCTCC-3’ | 5’-AGGCTCCAAATATAGGGGCAGGGTC-3’ |
|  | Col1a2 | 5’-CACCCCAGCGAAGAACTCATA-3’ | 5’-GCCACCATTGATAGTCTCTCCTAAC-3’ |
|  | IL-6 | 5’-TCTCTGCAAGAGACTTCCATCC-3’ | 5’-AGACAGGTCTGTTGGGAGTG-3’ |
|  | IL-1β | 5’-TGAGCTGAAAGCTCTCCACC-3’ | 5’-CTGATGTACCAGTTGGGGAA-3’ |
|  | CD163 | 5’-ATGGGCTAACTCCAGCGCCG-3’ | 5’-GATCCATCTGAGCAGGTCACTCCA-3’ |
